# Saffron is a monomorphic species as revealed by RAPD, ISSR and microsatellite analyses

**DOI:** 10.1186/1756-0500-2-189

**Published:** 2009-09-23

**Authors:** Angela Rubio-Moraga, Raquel Castillo-López, Lourdes Gómez-Gómez, Oussama Ahrazem

**Affiliations:** 1 Departamento de Ciencia y Tecnología Agroforestal y Genética. ETSIA. Universidad de Castilla-La Mancha, Campus Universitario s/n, Albacete, 02071. Spain; 2 VITAB Laboratorios. Polígono Industrial Garysol C/Pino, parcela 53, La Gineta, 02110, Albacete. Spain

## Abstract

**Background:**

Saffron (*Crocus sativus*) is considered the world's most expensive spice. Used mainly as a colorant for foodstuffs, it is highly appreciated for its aromatic and flavouring properties. Since no molecular markers for this species have been found in the literature, the objective of this study was to determine whether phenotypical differences found in *C. sativus *were supported by molecular analyses.

**Findings:**

Thirty primers from Operon Technologies were used in random amplified polymorphic DNA (RAPD) analysis, forty eight primers were screened using intersimple sequence repeats (ISSR) method and fifteen primers derived from a microsatellites library flanking sequences with repeat motifs were assayed in forty three isolates of *C. sativus *from eleven different countries and a *C. kotschyanus *isolate was used as outgroup. No polymorphic bands were detected in any of the accessions combining the different approaches used in this study.

**Conclusion:**

According to our findings, all accessions appear identical clones, not only because morphological characters but also at a molecular level. These data strongly suggested that *C. sativus *is a monomorphic species. Thus, genome sequencing is needed to find molecular markers for saffron.

## Background

The domesticated saffron (*Crocus sativus*) is an autumn-flowering perennial plant unknown in the wild. It is a sterile triploid form, possibly of the eastern Mediterranean autumn-flowering *C. cartwrightianus*, which originated in Crete, or in Central Asia. Mainly used as a colorant for foodstuffs, it is characterized by a bitter taste and a hay-like fragrance; due to the presence of picrocrocin and safranal. It also contains apocarotenoid compounds, which give food the yellow-orange color [1]. These traits make saffron a much-sought ingredient in many foods worldwide. Saffron was also said to have a high valued as medicinal plant, traditionally it used against cancer, depressive mood, menstruation disorders, liver disease and pain [2].

Today, saffron is cultivated from the Western Mediterranean (Spain) through Persia to India, Tibet and China. New cultivations have been created in Australia, Mexico, Argentine and New Zealand. Spain and Iran are the largest producers, together accounting for more than 80% of world production, which is approximately 205 tons per year [2].

The new Common Agricultural Policy of the European Union (CAP), along with increasing consumer demand for natural products of high quality, has led to resurgence in saffron culture after a long period of gradual decline and abandonment in traditional European production. Only 3,378 kg of saffron, with a value of approximately 4 million Euros, were produced in Spain in 2003. In spite of the reduced area dedicated to saffron cultivation, the employment generated by this sector is very high.

The planting of corms is a difficult task being planted one by one by hand. After a period of dormancy through the summer, the corms sprout in early autumn and flowers appear in mid-autumn. Flower harvest and removal of saffron stigmas are also mainly done by hand [2].

The taxonomy of *Crocus *is extremely complicated due to the lack of clear distinctive characters, the wide range of habitats and the heterogeneity of the morphological traits and cytological data. Phenotypical differences have been mentioned concerning the size and the number of the flowers produced by corms, although no molecular makers have been described for *Crocus sativus *[3].

The aim of this work was to determine whether *C. sativus *is a monomorphic or a polymorphic species. The different molecular approaches used were based on PCR methods such as random amplified polymorphic DNA (RAPD), intersimple sequence repeats (ISSR) and microsatellites, which have been widely used as markers [4-11].

## Methods

### Source of experimental material

For this study, 44 *Crocus sativus *isolates were included; with *C. kotschyanus *used as outgroup. The details of the accessions and their geographic origin are listed in Table 1. Three individuals representing each population were used. Plant tissues were independently harvested, frozen in liquid nitrogen and stored at -80°C until required.

**Table 1:** Geographic origin and accessions used in this study.

**Species**	**Geographic location**
*Crocus sativus*	Guadalajara, Spain
*Crocus sativus*	Gineta, Spain
*Crocus sativus*	El Bonillo, Spain
*Crocus sativus*	Zulema, Spain
*Crocus sativus*	Tobarra, Spain
*Crocus sativus*	Motilla del Palancar, Spain
*Crocus sativus*	Nava de Abajo, Spain
*Crocus sativus*	Abengibre, Spain
*Crocus sativus*	Fuentealbilla, Spain
*Crocus sativus*	Cordovilla, Spain
*Crocus sativus*	Lezuza, Spain
*Crocus sativus*	Ledaña, Spain
*Crocus sativus*	Alcala de Jucar, Spain
*Crocus sativus*	Tarazona de la Mancha, Spain
*Crocus sativus*	Minaya, Spain
*Crocus sativus*	Munera, Spain
*Crocus sativus*	Madridejos, Spain
*Crocus sativus*	La Solana, Spain
*Crocus sativus*	Madrigueras, Spain
*Crocus sativus*	Teruel, Spain
*Crocus sativus*	Pedroñeras, Spain
*Crocus sativus*	North region of Torbat, Iran
*Crocus sativus*	Central region of Torbat, Iran
*Crocus sativus*	Central region of Torbat, Iran
*Crocus sativus*	Central region of Torbat, Iran
*Crocus sativus*	Central region of Torbat, Iran
*Crocus sativus*	East region of Torbat, Iran
*Crocus sativus*	East region of Torbat, Iran
*Crocus sativus*	South east region of Torbat, Iran
*Crocus sativus*	East region of Ferdows, Iran
*Crocus sativus*	Italy
*Crocus sativus*	Italy
*Crocus sativus*	Italy
*Crocus sativus*	Azerbaijan
*Crocus sativus*	Azerbaijan
*Crocus sativus*	UK
*Crocus sativus*	UK
*Crocus sativus*	Turkey
*Crocus sativus*	Turkey
*Crocus sativus*	Taliouine, Morroco
*Crocus sativus*	Christchurch, New Zealand
*Crocus sativus*	Kashmir, India
*Crocus sativus*	China
*Crocus sativus*	Sierre de Cordoba, Argentine
*Crocus kotschyanus*	Pottertons nursery, UK

### DNA extraction

DNA was extracted from 150 to 300 mg of leaf material using a modified Doyle and Doyle method [12]. Leaf material was ground to a fine powder in liquid nitrogen and placed in a microcentrifuge tube with 2 mL of extraction buffer (2% CTAB, 100 mM Tris-HCl pH 8.0, 20 mM EDTA, 1.4 M NaCl, and 0.01% proteinase K) plus 40 μl of 2-mercaptoethanol. Following incubation at 65°C for 30 min, 1.4 mL of chloroform:isoamyl alcohol (24:1) was added, mixed and centrifuged at 8000 rpm for 30 min; the supernatant was transferred to a new tube and then repeated three times. DNA was precipitated with isopropanol (2/3 volume of supernatant), then centrifuged at 8000 rpm for 30 min, the supernatant discarded and the pellet washed in 70% ethanol containing 10 mM ammonium acetate for 20 min. The pellet was dissolved in 100 μL of TE buffer (10 mM Tris-HCl pH 7.4, 1 mM EDTA) and the DNA was reprecipitated with 1/2 volume of ammonium acetate 3 M and 2.5 volumes of ethanol. After centrifuging at 8000 rpm for 30 min, the pellet was redissolved in TE buffer with 10 μg/mL RNase and incubated at 30°C for 30 min. The extracted DNA was quantified with a spectrophotometer and diluted to 30 ng/μL in TE. The DNA was stored at -20°C for further analyses.

### Construction of a microsatellite library

A library of microsatellite repeats was constructed using a procedure described by Glen and Schable [13]. Briefly, genomic DNA (6 μg) was digested with restriction enzyme *Rsa*I and ligated with forward and reverse SuperSNX24 adaptors (SuperSNX24 forward 5'-GTTTAAGGCCTAGCTAGCAGCAGAATC and SuperSNX24 reverse 5'-GATTCTGCTAGCTAGGCCTTAAACAAAA). The restriction-ligation product was purified (wizard SV gel, Promega), and hybridized with biotinylated di- and trinucleotide-specific oligonucleotides [(TC)_9_, (TCC)_6_, (TGG)_6_, (TCT)_10_]. Hybridized fragments were captured twice with streptavidin-coated magnetic beads, and then amplified with adaptor-specific primers. The PCR products were ligated into a pGEMT (Promega, Madison, WI) and selected colonies were amplified and sequenced (ABI prism 310, Perkin Elmer). The microsatellites library was searched for sequences containing SSRs using web sat software [14], available at 

### DNA amplification

#### RAPD analysis

15 and 30 ng of genomic DNA were amplified in a volume of 20 μl containing 10 mM Tris-HCl pH 9.0, 2.5 mM MgCl_2_, 200 μM each dATP, dCTP, dGTP, dTTP, 0.4 μM primer, and 1 unit of Taq DNA polymerase by means of a thermal cycler (MJ-Mini, BioRad). The cycling programme began with an initial 2 min at 94°C followed by 45 cycles at 94°C for 30 s, 38°C for 30 s and 72°C for 2 min plus a final 10 min at 72°C and storage at 4°C. The sequences of primers are shown in additional file 1.

#### ISSR and microsatellite analyses

15 and 30 ng of genomic DNA were amplified in a volume of 25 μl containing 10 mM Tris-HCl pH 9.0, 1.5 mM MgCl_2_, 200 μM each dATP, dCTP, dGTP, dTTP, 0.4 μM primer, and 1 unit of Taq DNA polymerase by means of a thermal cycler (MJ-Mini, BioRad). The cycling programme began with an initial 2 min at 94°C followed by 45 cycles at 94°C for 45 s, 48-62°C for 45 s and 72°C for 2 min plus a final 10 min at 72°C and storage at 4°C. Primer sequences are shown in Additional file 2 and 3. A negative control was added in each run to test contamination in all analyses.

Amplification products were separated by electrophoresis in 2% agarose gel containing 1 μg/mL ethidium bromide and TAE buffer. Alternatively, samples from microsatellites amplification were separated by electrophoresis in 4% super-fine agarose or in 6% nondenaturing polyacrylamide gel. Ten microliters of amplified DNA were mixed with 3 μl sample buffer (1.2 mg/mL; 125 mg/mL Ficoll) and 10 μl was applied in each well of the gel. DNA molecular weight markers (1 kb, Promega, Madison, WI) were then added to each gel. The gels were run at a current of 50 mA until the bromophenol had migrated 10 cm from the well. The bands were then visualized under UV light and photographed. To confirm the data obtained from microsatellites sequencing analysis were performed.

## Results

gDNAs obtained from individual accessions (Spain (20), Iran (9), Italy (3), Azerbaijan (2), United Kingdom (2), Turkey (2), Morocco (1), New Zealand (1), India (1), Argentine (1) and China (1)) were used as template to perform the analyses using RAPD, ISSR and microsatellites methods.

**Figure 1:**
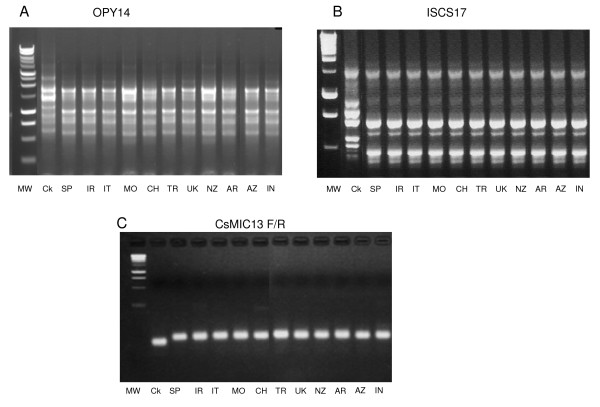
**Bands patterns of individual gDNA representing different geographic areas**. A. RAPD profile using OPY14 primer, B. ISSR profile using ISCS17 and C. Microsatellites profiles using CsMIC13 F and CSMIC13R. MW: Molecular weight (1 kb, Promega); Ck: *Crocus kotschyanus*; SP: *C. sativus *from Spain; IR: *C. sativus *from Iran; IT: *C. sativus *from Italy; MO: *C. sativus *from Morocco; CH: *C. sativus *from China; TR: *C. sativus *from Turkey; UK: *C. sativus *from United Kingdom; NZ: *C. sativus *from New Zealand; AR: *C. sativus *from Argentine; AZ: *C. sativus *from Azerbaijan and IN: *C. sativus *from India.

For RAPD analysis, from the 140 primers obtained from Operon Technologies (Huntsville, Alabama) and initially tested, only 30 primers were selected for further analysis. Of the 91 primers used in a first attempt at ISSR methods; 48 primers were selected (Additional file 1 and 2). The primers chosen for both methods produced distinctly robust and reproducible bands varying in numbers. While the remainder primers failed to produce bands or the profiles obtained were different between repeated experiments. For any of the primers selected, band scores did not differ between repeated experiments or between gels. A representative result of RAPD and ISSR profiles from each geographic area is shown in Figure 1A and 1B. All the accessions revealed the same band patterns whereas *C. kotschyanus *was different.

From the analysis of the microsatellite library. Forty seven of the 157 clones sequenced (31.3%) contained microsatellites. Repeat motifs and expected size are shown in Additional file 2. Sequences with repetitive motifs ranging from one to six were found. The number of repetitions ranged from 2 to 30, with di and tri-nucleotide repetitions found to be most abundant in the sequences screened. Two different di-nucleotide repetitions were found (AG/TC) and (CT/GA), representing 22% and 27% respectively for total repetition motifs, while 7 tri-nucleotide forms were detected (TCT/AGA), (CTT/GAA), (AAG/TTC), representing 8.5%, 8.5% and 6.2% respectively for total repetition motifs and with a 2% (AGC/TCG), (CAC/GTG), (CTC/GAG) and (CCT/GGA).

Primers were designed for the flanking sequences containing the repetition motif (Additional file 3). The expected size ranged from 148 pb for CSMIC23F/R and 372 bp for CSMIC19F/R. Fifteen of the primers designed were able to produce an amplification product, while no amplification was found when using the remaining primers. Nor did the profile patterns vary by changing the PCR conditions. The unsuccessful amplification in microsatellites analysis may be due to two possible reasons: in some cases the flanking regions were very short and we were unable to design good primers, in other cases we observed that in sequences containing large repetitive region polymerase enzymes failed to led amplification and may be explained by the formation of loops in the repetition regions. Bands obtained in microsatellites analysis were sequenced and no variations were observed. As happened with the RAPD and ISSR analyses, no polymorphic bands were encountered between the isolates of *C. sativus *whereas *C. kotschyanus *presented different size bands and different sequences with all the primers assayed. A representative result of microsatellites profiles from each geographic area is shown in Figure 1C.

## Discussion

In this study, three different PCR-based approaches: RAPD, ISSR and microsatellites were carried out in order to determine the variability of saffron with different geographic origins ranging from New Zealand to Spain.

A total of 140, 91 and 47 primers were used in RAPD, ISSR and microsatellites analysis, respectively. Only 30, 48 and 15 primers were able to produce reproducible bands in all the different accessions of *C. sativus *tested. No polymorphic bands were obtained in any accessions using the three methods, which strongly suggested that *C. sativus *is a monomorphic species. According to the results obtained in this study, all accessions appear identical clones, not only because of morphological characters but also at the molecular level, these results agree with those obtained by Grilli-Caiola et al. using RAPD analysis [15]. In addition, the existence of differences at the phenotypic level such as size of the flowers, the shape of the tepals, differences of colour and intensity in the tepals of samples collected from different origins could not be corroborated by molecular analysis,(data not shown), thus confirming the results previously obtained by Grilli-Caiola [16] using flow citometry.

*C. sativus*, as a sterile plant, fails to produce viable seeds and is thus dependent on human assistance. It seems to have undergone artificial selection in the past, a practice which offers advantages in maintaining its genetic characteristics while drastically reducing the variability of plants. Observed differences in saffron quality are mainly due to the methodology followed in the processing of stigmas, independent of the species origin [17].

Perhaps by using next-generation DNA sequencing methods introduced by the 454 Company in 2003, followed by Solexa and Solid techniques from other biotechnology companies, we can look more closely at the way in which saffron has evolved. Whole genome sequences could provide us with an opportunity to study saffron population genetics in order to find ways to discriminate between different isolates.

## Conclusion

Three PCR-based approaches: RAPD, ISSR and microsatellites failed to find polymorphisms in saffron collected from different origins. Our data suggest that saffron is a monomorphic species. To find the way to discriminate between isolates of *C. sativus *whole genome sequences is needed.

## Competing interests

The authors declare that they have no competing interests.

## Authors' contributions

ARM collected the data and participated in its analysis; RCL participated in the analysis of the data; LGG participated in the design of the study and in the analysis of the data; OA participated in the design of the study, in the analysis of the data and wrote the manuscript. All authors were involved in the interpretation of the data and approved the final manuscript.

## Supplementary Material

Additional file 1 Primers sequences used in RAPD analysis. -: negative amplification or no reproducible patterns; +: positive amplification. Click here for file

Additional file 2 Primers sequences and annealing temperature (Tm) used in ISSR analysis. Click here for file

Additional file 3 Primers sequences, repeat motif, annealing temperature (Tm) and size expected in microsatellites analysis. Click here for file
