# Supplementary material for: Saffron is a monomorphic species as revealed by RAPD, ISSR and microsatellite analyses
Source: BMC Res Notes. 2009 Sep 23;2:189. doi: 10.1186/1756-0500-2-189 (PMC2758891; doi:10.1186/1756-0500-2-189)
Supplement: Additional file 1 — Primers sequences used in RAPD analysis. -: negative amplification or no reproducible patterns; +: positive amplification. [file 1756-0500-2-189-S1.DOC]

Additional file 1. Primers sequences used in RAPD analysis. -: negative amplification or no reproducible patterns; +: positive amplification

| PRIMER NAME | SEQUENCE | Amplification | PRIMER NAME | SEQUENCE | Amplification |
| --- | --- | --- | --- | --- | --- |
| OPA-01 | CAGGCCCTTC | + | OPJ-01 | CCCGGCATAA | - |
| OPA-02 | TGCCGAGCTG | - | OPJ-02 | CCCGTTGGGA | - |
| OPA-03 | AGTCAGCCAC | - | OPJ-03 | TCTCCGCTTG | - |
| OPA-04 | AATCGGGCTG | + | OPJ-04 | CCGAACACGG | - |
| OPA-05 | AGGGGTCTTG | - | OPJ-05 | CTCCATGGGG | - |
| OPA-06 | GGTCCCTGAC | - | OPJ-06 | TCGTTCCGCA | - |
| OPA-07 | GAAACGGGTG | - | OPJ-07 | CCTCTCGACA | - |
| OPA-08 | GTGACGTAGG | - | OPJ-08 | CATACCGTGG | - |
| OPA-09 | GGGTAACGCC | + | OPJ-09 | TGAGCCTCAC | - |
| OPA-10 | GTGATCGCAG | - | OPJ-10 | AAGCCCGAGG | - |
| OPA-11 | CAATCGCCGT | - | OPJ-11 | ACTCCTGCGA | - |
| OPA-12 | TCGGCGATAG | - | OPJ-12 | GTCCCGTGGT | - |
| OPA-13 | CAGCACCCAC | - | OPJ-13 | CCACACTACC | + |
| OPA-14 | TCTGTGCTGG | - | OPJ-14 | CACCCGGATG | - |
| OPA-15 | TTCCGAACCC | - | OPJ-15 | TGTAGCAGGG | - |
| OPA-16 | AGCCAGCGAA | - | OPJ-16 | CTGCTTAGGG | - |
| OPA-17 | GACCGCTTGT | - | OPJ-17 | ACGCCAGTTC | - |
| OPA-18 | AGGTGACCGT | - | OPJ-18 | TGGTCGCAGA | - |
| OPA-19 | CAAACGTCGG | - | OPJ-19 | GGACACCACT | - |
| OPA-20 | GTTGCGATCC | - | OPJ-20 | AAGCGGCCTC | - |

| PRIMER NAME | SEQUENCE | Amplification | PRIMER NAME | SEQUENCE | Amplification |
| --- | --- | --- | --- | --- | --- |
| OPK-01 | CATTCGAGCC | - | OPL-01 | GGCATGACCT | - |
| OPK-02 | GTCTCCGCAA | - | OPL-02 | TGGGCGTCAA | - |
| OPK-03 | CCAGCTTAGG | - | OPL-03 | CCAGCAGCTT | - |
| OPK-04 | CCGCCCAAAC | - | OPL-04 | GACTGCACAC | - |
| OPK-05 | TCTGTCGAGG | - | OPL-05 | ACGCAGGCAC | - |
| OPK-06 | CACCTTTCCC | - | OPL-06 | GAGGGAAGAG | - |
| OPK-07 | AGCGAGCAAG | - | OPL-07 | AGGCGGGAAC | + |
| OPK-08 | GAACACTGGG | + | OPL-08 | AGCAGGTGGA | - |
| OPK-09 | CCCTACCGAC | + | OPL-09 | TGCGAGAGTC | - |
| OPK-10 | GTGCAACGTG | - | OPL-10 | TGGGAGATGG | - |
| OPK-11 | AATGCCCCAG | - | OPL-11 | ACGATGAGCC | + |
| OPK-12 | TGGCCCTCAC | - | OPL-12 | GGGCGGTACT | - |
| OPK-13 | GGTTGTACCC | - | OPL-13 | ACCGCCTGCT | - |
| OPK-14 | CCCGCTACAC | - | OPL-14 | GTGACAGGCT | - |
| OPK-15 | CTCCTGCCAA | + | OPL-15 | AAGAGAGGGG | - |
| OPK-16 | GAGCGTCGAA | + | OPL-16 | AGGTTGCAGG | + |
| OPK-17 | CCCAGCTGTG | + | OPL-17 | AGCCTGAGCC | + |
| OPK-18 | CCTAGTCGAG | - | OPL-18 | ACCACCCACC | + |
| OPK-19 | CACAGGCGGA | - | OPL-19 | GAGTGGTGAC | + |
| OPK-20 | GTGTCGCGAG | - | OPL-20 | TGGTGGACCA | + |

| PRIMER NAME | SEQUENCE | Amplification | PRIMER NAME | SEQUENCE | Amplification |
| --- | --- | --- | --- | --- | --- |
| OPR-01 | TGCGGGTCCT | - | OPY-01 | GTGGCATCTC | + |
| OPR-02 | CACAGCTGCC | - | OPY-02 | CATCGCCGCA | + |
| OPR-03 | ACACAGAGGG | - | OPY-03 | ACAGCCTGCT |  |
| OPR-04 | CCCGTAGCAC | - | OPY-04 | GGCTGCAATG | + |
| OPR-05 | GACCTAGTGG | + | OPY-05 | GGCTGCGACA | - |
| OPR-06 | GTCTACGGCA | + | OPY-06 | AAGGCTCACC | - |
| OPR-07 | ACTGGCCTGA | + | OPY-07 | AGAGCCGTCA | - |
| OPR-08 | CCCGTTGCCT | - | OPY-08 | AGGCAGAGCA | - |
| OPR-09 | TGAGCACGAG | - | OPY-09 | AGCAGCGCAC | - |
| OPR-10 | CCATTCCCCA | + | OPY-10 | CAAACGTGGG | - |
| OPR-11 | GTAGCCGTCT | - | OPY-11 | AGACGATGGG | - |
| OPR-12 | ACAGGTGCGT | - | OPY-12 | AAGCCTGCGA | - |
| OPR-13 | GGACGACAAG | - | OPY-13 | GGGTCTCGGT | - |
| OPR-14 | CAGGATTCCC | - | OPY-14 | GGTCGATCTG | + |
| OPR-15 | GGACAACGAG | + | OPY-15 | AGTCGCCCTT | + |
| OPR-16 | CTCTGCGCGT | - | OPY-16 | GGGCCAATGT | + |
| OPR-17 | CCGTACGTAG | - | OPY-17 | GACGTGGTGA | + |
| OPR-18 | GGCTTTGCCA | - | OPY-18 | GTGGAGTCAG | + |
| OPR-19 | CCTCCTCATC | - | OPY-19 | TGAGGGTCCC | - |
| OPR-20 | ACGGCAAGGA | - | OPY-20 | AGCCGTGGAA | + |
