# Supplementary material for: Saffron is a monomorphic species as revealed by RAPD, ISSR and microsatellite analyses
Source: BMC Res Notes. 2009 Sep 23;2:189. doi: 10.1186/1756-0500-2-189 (PMC2758891; doi:10.1186/1756-0500-2-189)
Supplement: Additional file 2 — Primers sequences and annealing temperature (Tm) used in ISSR analysis. [file 1756-0500-2-189-S2.DOC]

Additional file 2. Primers sequences and annealing temperature (Tm) used in ISSR analysis.

| Primers | Sequence (5’→3’) | Tm(ºC) | Amplification |
| --- | --- | --- | --- |
| ISCS1 | TCCTCCTCCTCCTCCTCCC | 58 | + |
| ISCS2 | GATGATGATGATGATGATG | 53 | - |
| ISCS3 | CTTCTTCTTCTTCTTCTTCTTC | 54 | - |
| ISCS4 | TGGTGGTGGTGGTGGTGGC | 58 | - |
| ISCS5 | GTGTGTGTGTGTGTGTGTG | 50 | - |
| ISCS6 | GAGAGAGAGAGAGAGAG | 48 | - |
| ISCS7 | TCTCTCTCTCTCTCTCTCC | 50 | + |
| ISCS8 | TCTTCTTCTTCTTCTTCTTCTTCTTCTA | 48 | + |
| ISCS9 | ATCATCATCATCATCATCATCATCATCATCG | 62 | + |
| ISCS10 | ACACACACACACACACC | 50 | + |
| ISCS11 | CTCTCTCTCTCTCTCTT | 48 | + |
| ISCS12 | TTGTTGTTGTTGTTGTTGC | 55 | + |
| ISCS13 | TATTATTATTATTATTATTATTATG | 50 | - |
| ISCS14 | AGTGAGTGAGTGAGTGAGTGA | 52 | - |
| ISCS15 | TATCTATCTATCTATCTATCTATCT | 48 | + |
| ISCS16 | HBHGAGGAGGAGGAGGAG | 59 | - |
| ISCS17 | DBDBCACCACCACCACCAC | 61 | + |
| ISCS18 | DBDBCCACCACCACCACCA | 62 | + |
| ISCS19 | HVHGTGGTGGTGGTGGTG | 61 | + |
| ISCS20 | DHBCGACGACGACGACGA | 62 | + |
| ISCS21 | BDBACAACAACAACAACA | 50 | + |
| ISCS22 | HBBGAAGAAGAAGAAGAA | 48 | + |
| ISCS23 | HBDBGACCGACCGACCGACC | 62 | + |
| ISCS24 | HBVBGATAGATAGATAGATA | 48 | - |
| ISCS25 | HBVCGATCGATCGATCGAT | 50 | - |
| ISCS26 | GTGTGTGTGTGTGTGTYG | 52 | + |
| ISCS27 | TCTCTCTCTCTCTCTCRA | 48 | + |
| ISCS28 | TCTCTCTCTCTCTCTCRT | 48 | + |
| ISCS29 | TCTCTCTCTCTCTCTCRG | 50 | + |
| ISCS30 | ACACACACACACACACYT | 50 | + |
| ISCS31 | ACACACACACACACACYA | 48 | - |
| ISCS32 | ACACACACACACACACYG | 52 | + |
| ISCS33 | TGTGTGTGTGTGTGTGRT | 52 | + |
| ISCS34 | TGTGTGTGTGTGTGTGRC | 52 | + |
| ISCS35 | TGTGTGTGTGTGTGTGRA | 50 | + |
| ISCS36 | ACCACCACCACCACCACC | 62 | - |
| ISCS37 | AGCAGCAGCAGCAGCAGC | 62 | - |
| ISCS38 | GTGTGTGTGTGTGTGTYC | 50 | + |
| ISCS39 | ATGATGATGATGATGATG | 50 | - |
| ISCS40 | CCGCCGCCGCCGCCGCCG | 60 | - |
| ISCS41 | CTCCTCCTCCTCCTCCTC | 60 | - |
| ISCS42 | GGCGGCGGCGGCGGCGGC | 60 | - |
| ISCS43 | GAAGAAGAAGAAGAAGAA | 50 | + |
| ISCS44 | GTTGTTGTTGTTGTTGTT | 50 | - |
| ISCS45 | TGCTGCTGCTGCTGCTGC | 60 | - |
| ISCS46 | GTGTGTGTGTGTGTGTYA | 50 | + |
| ISCS47 | CACACACACACACACARG | 52 | + |
| ISCS48 | GACAGACAGACAGACA | 48 | + |
| ISCS49 | CCCTCCCTCCCTCCCT | 60 | + |
| ISCS50 | CACACACACACACACARC | 52 | + |
| ISCS51 | CACACACACACACACART | 50 | + |
| ISCS52 | TGCATGCATGCATGCA | 62 | - |
| ISCS53 | GGATGGATGGATGGAT | 48 | - |
| ISCS54 | CTCTCTCTCTCTCTCTRG | 48 | + |
| ISCS55 | CTCTCTCTCTCTCTCTRC | 48 | + |
| ISCS56 | CTCTCTCTCTCTCTCTRA | 48 | - |
| ISCS57 | GAGAGAGAGAGAGAGAYG | 48 | + |
| ISCS58 | GAGAGAGAGAGAGAGAYC | 48 | + |
| ISCS59 | GAGAGAGAGAGAGAGAYT | 48 | - |
| ISCS60 | AGAGAGAGAGAGAGAGT | 48 | - |
| ISCS61 | AGAGAGAGAGAGAGAGC | 48 | - |
| ISCS62 | AGAGAGAGAGAGAGAGG | 48 | - |
| ISCS63 | GAGAGAGAGAGAGAGAT | 48 | - |
| ISCS64 | GAGAGAGAGAGAGAGAC | 48 | + |
| ISCS65 | GAGAGAGAGAGAGAGAA | 48 | + |
| ISCS66 | CTCTCTCTCTCTCTCTA | 48 | - |
| ISCS67 | CTCTCTCTCTCTCTCTG | 48 | - |
| ISCS68 | CACACACACACACACAT | 50 | - |
| ISCS69 | CACACACACACACACAA | 52 | + |
| ISCS70 | CACACACACACACACAG | 52 | + |
| ISCS71 | GTGTGTGTGTGTGTGTA | 48 | - |
| ISCS72 | GTGTGTGTGTGTGTGTC | 50 | + |
| ISCS73 | GTGTGTGTGTGTGTGTT | 50 | + |
| ISCS74 | TCTCTCTCTCTCTCTCA | 48 | - |
| ISCS75 | TCTCTCTCTCTCTCTCC | 48 | - |
| ISCS76 | TCTCTCTCTCTCTCTCG | 50 | + |
| ISCS77 | ACACACACACACACACT | 50 | + |
| ISCS78 | ACACACACACACACACG | 52 | - |
| ISCS79 | TGTGTGTGTGTGTGTGA | 52 | + |
| ISCS80 | TGTGTGTGTGTGTGTGC | 50 | + |
| ISCS81 | TGTGTGTGTGTGTGTGG | 50 | + |
| ISCS82 | ATATATATATATATATYA | 50 | - |
| ISCS83 | ATATATATATATATATYC | 48 | - |
| ISCS84 | ATATATATATATATATYG | 48 | - |
| ISCS85 | AGAGAGAGAGAGAGAGYT | 50 | - |
| ISCS86 | AGAGAGAGAGAGAGAGYC | 50 | - |
| ISCS87 | AGAGAGAGAGAGAGAGYA | 50 | + |
| ISCS89 | TATATATATATATATART | 50 | - |
| ISCS90 | TATATATATATATATARC | 48 | - |
| ISCS91 | TATATATATATATATARG | 48 | - |

-: negative amplification; +: positive amplification
