# Supplementary material for: Saffron is a monomorphic species as revealed by RAPD, ISSR and microsatellite analyses
Source: BMC Res Notes. 2009 Sep 23;2:189. doi: 10.1186/1756-0500-2-189 (PMC2758891; doi:10.1186/1756-0500-2-189)
Supplement: Additional file 3 — Primers sequences, repeat motif, annealing temperature (Tm) and size expected in microsatellites analysis. [file 1756-0500-2-189-S3.DOC]

Additional file 3. Primers sequences, repeat motif, annealing temperature (Tm) and size expected in microsatellites analysis.

| Primers | Sequence (5’→3’) | Repeat motif | Tm (ºC) | Amplification | Size (bp) |
| --- | --- | --- | --- | --- | --- |
| CSMIC10F CSMIC10R | AATCACACACAACATGGTCGTT  TGTTTAACCCAGCTAGCAGAAT | (GA)_16_ (GAAGA)_2_ (ATCAC)_2_ | 52 | - | 298 |
| CSMIC13F CSMIC13R | TGGCATTAGATTACGGGTTTGT  GAATCACGTTGTTGGGATTGAT | (CT)_14_ | 57 | + | 195 |
| CSMIC14F CSMIC14R | CCTTGTCTTGAACGAATGTCTG  TTGCAGAATCCTTGGCCTTA | (T)_11_ (TCTTCC)_2_ (T)_13_ | 60 | + | 285 |
| CSMIC15F CSMIC15R | AATTTGACCGTTGGATAACACC  AATCACTTCTCTCTGCCGATG | (CCCATC)_2_ (GA)_9_ (AAG)_5_ | 57 | - | 278 |
| CSMIC19F CSMIC19R | GGCCTAGCTAGCAGAATCACAA  AGCTAGCAGAATCACACTCTT | (AAGTA)_2_ (AGTA)_3_ (TCAAG)_2_ (CCATGT)_2_ (AGTA)_3_ | 57 | - | 372 |
| CSMIC20F CSMIC20R | GCTAGCAGAATCACAATGGTTA  ACCTGGAGGAGCAGCAGTAA | (TCT)_6_ | 51 | - | 154 |
| CSMIC21F CSMIC21R | CTCGCTAGCCGAATCACAACT  TGTGAGAGCACACGGTGT | (GA)_30_ | 50 | - | 200 |
| CSMIC23F CSMIC23R | GTCACTTACATGTTGGTGT  AATTCTATTCCAAGGCTCCA | (AAG)_5_ (TCATA)_2_ | 50 | + | 148 |
| CSMIC25F CSMIC25R | GTCTCCTTCGCTATCTCCTTGA  ACCTTCAAGAAGATCAGCAAT | (TCT)_5_ | 50 | + | 134 |
| CSMIC26F CSMIC26R | ATCACTCATAACTCTCCATGA  AGCTAGCAGATCACATAGGT | (CTT)_4_ (TCTTC)_2_ | 53 | - | 313 |
| CSMIC27F CSMIC27R | TGGATATACGTAATCCAGACT  ATAATTCCTGAGGTGAATT | (AAACT)_2_ (CCTTGC)_2_ (CCCAA)_2_ | 50 | - | 297 |
| CSMIC28F CSMIC28R | TAAGGCCATGCTAGCAGAAT  AGCAGTAATTCGTAGCGACA | (CTC)_5_ (TTC)_13_ (CTTCTG)_3_ (CTT)_7_ (TCT)_6_ (TCCTCT)_2_ | 48 | - | 305 |
| CSMIC29F  CSMIC29R | TAGCAGAATCACAATTGAGCA  AGGCCTAGCTGCGGATCACCT | (CT)_17_ | 50 | - | 167 |
| CSMIC30F  CSMIC30R | GCAGAATCACACATGGTTACAA  AGTGACACGACACTCACTAT | (TC)_22_ | 50 | - | 259 |
| CSMIC31F  CSMIC31R | TCAGGTACGACCGGTGTAA  CCGAAGTATGTCGCTGATCTTA | (TC)_15_, (CT)_5_ | 55 | - | 273 |
| CSMIC32F  CSMIC32R | AAGGCATAAGCTAGCAGAATCA  CTAGATCCACCGATCACAAT | (CCCTT)_2_ (CCCTTC)_2_ (CTCC)_4_ | 52 | - | 222 |
| CSMIC34F CSMIC34R | TGTAATCAGGACAGATCAAGGA  AGGCCTAGCTAGCAGAATCA | (GTCGTG)_3_ | 52 | - | 193 |
| CSMIC35F CSMIC35R | AGGCCTAGCTAGCAGAATCACT  GTGTCCAGATGAAGGCAGGCA | (T)_17_ (TATGA)_2_ (CTT)_5_ | 55 | - | 282 |
| CSMIC36F CSMIC36R | GCTAGCAGAATCACATGATCCA  AGTGCATTCATCTCACCTCTCA | (CAC)_5_ | 50 | + | 292 |
| CSMIC37F CSMIC37R | GAAGCAACAATGGCGGTGGA  TCGGAGCGGTGGAGATCGTCT | (TC)_21_ | 60 | + | 231 |
| CSMIC38F CSMIC38R | GTCTAAGGCCTAGCTAGCAGA  GTTATCAAATGTTGGCCCACT | (CT)_27_ | 58 | - | 304 |
| CSMIC39F CSMIC39R | GCTAGCAGAATCACTACTTGA  AATGTTGGCCCACTCACACT | (CT)_27_ | 54 | - | 344 |
| CSMIC40F CSMIC40R | TAGCAGAATCACATTGAATGCA  CCATGTGATGATGTGAGCAGCA | (TC)_10_ (TCGGGT)_2_ | 52 | - | 223 |
| CSMIC41F CSMIC41R | AGAGGAGGAGATTATCAAGA  ATACGCTGCACGTATATCAA | (AG)_17_ | 52 | - | 242 |
| CSMIC42F CSMIC42R | CAGAATCACTTACCAGGTCAGT  CGTGGTACAGTGTAGCTACTTA | (CTT)_9_ (TTCCT)_5_ (CTT)_9_ | 54 | + | 265 |
| CSMIC43F CSMIC43R | GCAGAATCACTACTTGAAGACA  TGAGATGGATATATTCTCTGA | (CT)_27_ | 52 | - | 334 |
| CSMIC44F CSMIC44R | CAGTGCTTCGGCTGAATGTGAA  ACTGCTGGACGGTGCAACTT | GCTG)_4_ (CCT)_12_ (TTTTC)_2_ | 60 | + | 217 |
| CSMIC45F CSMIC45R | CCGCCTAGCTAGCAGAATCACA  GATAAGACCTGCAACTTCAACT | (CT)_10_ | 52 | - | 167 |
| CSMIC46F CSMIC46R | GTACAGTGCTGAAGAGGAGGA  TGGATACGCTGCACGTATCTCA | (AG)_18_ | 58 | + | 227 |
| CSMIC47F CSMIC47R | ACCAGGTCAGTTGATGCCTCAT  CAGTGTAGCTACTTAGACAGT | (CTT)_9_ (TTCCT)_6_ (CTT)_9_ | 48 | + | 253 |
| CSMIC48F CSMIC48R | GCGAGCGAAATCACAATCTCGA  GCGAGCGAAATCACAATCTCGA | (TC)_19_ | 52 | - | 237 |
| CSMIC49F CSMIC49R | ACTAGTTCACTCATCCGTTA  TGAATCGAATGGGTAGGGAAT | (AG)_17_ | 50 | - | 197 |
| CSMIC50F CSMIC50R | TAACCTCGTCGGAGCGGTGGA  GGAGCAACAATGGCGGTGGAA | (AG)_21_ | 60 | + | 239 |
| CSMIC51F CSMIC51R | GACGGGTAGTAGAAAGTTCTTCA  CGAATGGGTCTCCAAACCCT | (AG)_27_ | 58 | - | 331 |
| CSMIC53F CSMIC53R | GCAGAATCACTGCTGGACGGGT  CAGTGCTTCGGCTGAATGTGAA | (AGC)_9_ | 62 | + | 216 |
| CSMIC54F CSMIC54R | AGCAGCAGAGAAGTAAGACAGT  TCAACTTCCCAACCACTTTGA | (GA)_10_ | 56 | - | 158 |
| CSMIC55F CSMIC55R | AGCAACAGAGGCACACATTCA  AGCTGTCAGTCCAATCATCAAC | (CTAT)_9_ | 60 | + | 268 |
| CSMIC56F CSMIC56R | CTTATTGGATACGCTGCA  TAAGCCTAGCTAGCAGAA | (CT)_18_ | 52 | - | 260 |
| CSMIC57F CSMIC57R | GTAACCTGCTCCAGTGCTA  TAAGGCCTAGCTAGCAGAATCT | (AAG)_5_ (AAG)_6_ (GAAAG)_6_ | 57 | - | 321 |
| CSMIC58F CSMIC58R | TCGTCATGGTCGTCGCTACTA  AGCCTAGCTAGCAGATCATAGA | (CCTCT)_3_ (CTCCC)_2_ (CTACT)_2_ (TTCGTC)_2_ | 58 | - | 259 |
| CSMIC59F CSMIC59R | GAATATTGTTGATGAGGCCGGA  AAGAGAGATATTAAATAAGTCGCA | (AG)_8_ (AG)_12_ | 55 | + | 196 |
| CSMIC60F CSMIC60R | CATCGGCCTGAATGCCGT  GGGAAGTTCAAATCCCACTA | (TC)_17_ | 58 | - | 191 |
| CSMIC61F CSMIC61R | TTCAAGTGCTTATTGGTCCA  CATGTTCAATGCTTCATCAAGT | (TC)_12_ | 58 | - | 318 |
| CSMIC62F CSMIC62R | CCAATCTGAGGACGGGCT  AGAAGCGTGATGAAGTGA | (GA)_8_ (AG)_7_ | 55 | + | 325 |
| CSMIC7F CSMIC7R | GAATCACCACCTGAATTGTGAG  AAGAGGTCGAAGAAGGGAAAAC | (TCT)_5_ | 50 | - | 283 |
| CSMIC8F CSMIC8R | TCTTGGAATGGTTAGAGCGTGT  ACCAGCAATCTTTGGAACAGTC | (GAGTT)_2_ (CGACC)_2_ | 51 | - | 298 |
| CSMIC9F CSMIC9R | ACTGAAAGAAAGGGGAGAAAGG  TATATCGAATGGAGGTTCCGTC | (TTAAA)_2_(CACTTT)_2_ | 50 | - | 235 |

-: negative amplification; +: positive amplification
